# Cost-effectiveness analysis of interventions for migraine in four low- and middle-income countries

**DOI:** 10.1186/s10194-015-0496-6

**Published:** 2015-02-18

**Authors:** Mattias Linde, Timothy J Steiner, Dan Chisholm

**Affiliations:** Department of Neuroscience, Norwegian University of Science and Technology, Trondheim, Norway; Norwegian Advisory Unit on Headaches, St. Olavs University Hospital, Nevrosenteret Øst, 7006 Trondheim, Norway; Division of Brain Sciences, Imperial College London, London, UK; Department of Mental Health and Substance Abuse, WHO, Geneva, Switzerland

**Keywords:** Analysis, Cost effectiveness, Drug therapy, Economics, Migraine, Public health

## Abstract

**Background:**

Evidence of the cost and effects of interventions for reducing the global burden of migraine remains scarce. Our objective was to estimate the population-level cost-effectiveness of evidence-based migraine interventions and their contributions towards reducing current burden in low- and middle-income countries.

**Methods:**

Using a standard WHO approach to cost-effectiveness analysis (CHOICE), we modelled core set intervention strategies for migraine, taking account of coverage and efficacy as well as non-adherence. The setting was primary health care including pharmacies. We modelled 26 intervention strategies implemented during 10 years. These included first-line acute and prophylactic drugs, and the expected consequences of adding consumer-education and provider-training. Total population-level costs and effectiveness (healthy life years [HLY] gained) were combined to form average and incremental cost-effectiveness ratios. We executed runs of the model for the general populations of China, India, Russia and Zambia.

**Results:**

Of the strategies considered, acute treatment of attacks with acetylsalicylic acid (ASA) was by far the most cost-effective and generated a HLY for less than US$ 100. Adding educational actions increased annual costs by 1–2 US cents per capita of the population. Cost-effectiveness ratios then became slightly less favourable but still less than US$ 100 per HLY gained for ASA. An incremental cost of > US$ 10,000 would have to be paid per extra HLY by adding a triptan in a stepped-care treatment paradigm. For prophylaxis, amitriptyline was more cost-effective than propranolol or topiramate.

**Conclusions:**

Self-management with simple analgesics was by far the most cost-effective strategy for migraine treatment in low- and middle-income countries and represents a highly efficient use of health resources. Consumer education and provider training are expected to accelerate progress towards desired levels of coverage and adherence, cost relatively little to implement, and can therefore be considered also economically attractive. Evidence-based interventions for migraine should have as much a claim on scarce health resources as those for other chronic, non-communicable conditions that impose a significant burden on societies.

## Background

Migraine is common in every part of the world [1]. The Global Burden of Disease study 2010 (GBD 2010) found it to be the third most prevalent disorder in the world and among the top ten causes of years of healthy life lost to disability (YLDs) [2]. Therapeutic options have increased greatly over the last decades, but are not universally available. Economic evaluation can play a critical role not only in identifying the most cost-effective migraine therapies but also in demonstrating how health-care resource allocation to migraine treatment might contribute to overall health gain.

The literature is unhelpful. A systematic review captured 21 cost-effectiveness analyses (CEAs) of migraine interventions, with wide variation in the methods used and results obtained [3]. Most studies (15/21) compared different triptans. None studied over-the-counter (OTC) drugs in monotherapy, which are by far the most used treatments in all countries. Only four studies examined the cost-effectiveness of prophylactic medications, mainly the antiepileptic drugs, and only one provided a comparison with the prevailingly-used beta-blockers [4]. None has looked beyond Europe and North America. Thus, the cost-effectiveness is unknown of the most commonly used drugs anywhere, and of all drugs in 90% of the world [5].

Our objective was to inform health policy by evaluating the cost-effectiveness of a selected core set of interventions for migraine in low- and middle-income countries.

## Methods

We modelled costs and effects at population level in four countries: three of the large, middle-income BRIC countries, Russia, India and China (excluding Brazil, for which we had insufficient data), and one lower middle-income country, Zambia, for comparison.

### Selection of interventions

We adopted a core set of drug interventions, focusing on those included in WHO’s Essential Medicines list [6-8] but also those backed by substantial evidence of effectiveness. We included first-line (simple analgesics, *eg*, acetylsalicylic acid [ASA] 1,000 mg, but not paracetamol, for lack of evidence) and second-line medications (sumatriptan 50 mg because of its low price compared to other triptans, and almotriptan 12.5 mg because it had earlier been signified as the most cost-effective triptan [9]) for acute treatment of attacks, and assumed the latter would be used only by non-responders to the former in a stepped-care treatment paradigm (Table 1). We also included prophylactic drugs (propranolol 160 mg, topiramate 100 mg, amitriptyline 100 mg daily). We followed guidelines for dosages [6,7]. We added to the model the expected consequences of consumer education (posters and leaflets in pharmacies explaining how to acquire and best use these medications) and of training of health-care providers.

**Table 1:** **Assumptions made, how they were justified and their impact or weight in the model**

	**Assumption**	**Justification**	**Impact or weight in the model**
1	Mild headache is not associated with disability	This was a standard assumption [10,2]	If the assumption were false, the cost-effectiveness of acute therapy would be slightly reduced
2	The pain associated with untreated migraine is at least moderate	On the one hand, the diagnostic criteria for migraine describe the pain as at least moderate [11]; on the other hand, most clinical trials have recruited patients with at least moderate pain	None
3	In a stepped-care treatment paradigm, triptans are used only by non-responders to simple analgesics	This is the standard stepped-care paradigm, in which more expensive medications are reserved for those shown to need them	The alternative would be a multiplicity of scenarios of no obvious interest
4	Acute treatment is initiated at attack onset (commencement of pain phase)	The assumption was necessary to establish a time zero for purposes of effect calculation and was subject to further assumptions regarding patient adherence (see below)	To the extent that the assumption was false, effect and therefore cost-effectiveness would be reduced
5	Each acute medication is used once per attack	The assumption was necessary because of dependence on clinical trials data	Additional doses would increase cost, particularly in the case of triptans (mean number of triptan doses per attack was reported as 1.4-1.5 in the USA [12], but this may not be representative of the countries of interest)
6	The endpoint of sustained headache-relief is an all-or-nothing response	The assumption is in line with the standard definitions of pain relief and sustained pain freedom [13]	The consequence of the assumption was an underestimation of effectiveness
7	Prophylaxis is offered only to the proportion of people with ≥3 migraine attacks/month	As a recommendation based on frequency only, this was conservatively chosen [6]	This is a clinical rather than an economic threshold, so it would be of limited interest to vary it. Lowering the threshold to ≥2 would increase the use of prophylactics with less gain per user
8	ASA has high current coverage (80%) in all study contexts except Zambia (50%)	This was conservative; ASA is available almost universally, but not easily in rural Zambia	No impact on cost-effectiveness estimations. Higher coverage would allow greater population health gain
9	As a result of non-adherence, a proportion of patients use OTC-drugs later than is ideal, and in suboptimal doses (described in the text).	Best estimate, formed from our clinical judgement	Better adherence would lead to higher health gain and therefore improve cost-effectiveness
10	Provider adherence is 75%	Best estimate, based on our experience	Higher adherence would allow greater population health gain, which would improve cost-effectiveness estimations
11	Public education improves adherence by 50% of the current deficit	Based on what can be in expected in real world settings	A greater improvement of consumer adherence would lead to improved cost-effectiveness as well as greater population health gain
12	Three-monthly doctor visits, each of 10 minutes’ duration, are needed for monitoring and prescription of triptans and prophylactics	Reflects typical clinical need and treatment practice in these countries	More or longer visits would increase costs
13	For consumer education, the number of leaflets needed is 50% of the disease prevalence, and one poster is required per 2,000 of the population	Leaflet numbers allows for high circulation/exposure; poster numbers conform to WHO programme costing standards	Increasing or decreasing leaflet or poster numbers would have a negligible impact on base-line results because the base-line cost of consumer education is very low (US$ 0.01-0.02 per capita)
14	For provider education, one physician per primary health-care centre per year will be trained for one day	This represents an effective approach to reaching primary health care throughout the country	Increasing or decreasing the number of trained providers would have a negligible impact on base-line results because the base-line cost of provider education is very low (US$ 0.01-0.02 per capita)

### Analytical model

We used the sectoral, population-based approach to cost-effectiveness analysis (CEA) and the methods and tools developed by WHO-CHOICE [14]. Specifically, we ran a population model for two scenarios over a lifetime analytical horizon (100 years) to give the total number of healthy years lived by the population. Scenario 1 represented the natural history of migraine (no interventions in place); scenario 2 reflected the population-level impact of each specified intervention implemented for 10 years (thereafter, epidemiological variables and health-state valuations returned to natural history values). The difference between these two simulations represented the population-level health gain (healthy life years [HLYs] gained) from the intervention, relative to doing nothing.

We applied separate disability weights (DWs) (health state valuations on a 0–1 scale, where 1 equals full health) to the times spent in the ictal (within-attack) state of migraine and the interictal state (between attacks, but susceptible). Ictal DW (0.43) was available from GBD 2010 [2]. For interictal DW we applied the lowest weighting of 0.01, and only to those with high-frequency attacks. These DWs were multiplied by the estimated amounts of time spent in an ictal or interictal state by persons with migraine, with and without intervention.

Analyses were limited to the population aged 18–65 years because neither efficacy nor epidemiological data were reliably available for other age groups.

### Epidemiological data

Sex-specific prevalences (Table 2) were drawn from epidemiological surveys performed in the four countries as projects within the Global Campaign against Headache by *Lifting The Burden* (LTB) [11,15-18]. The same sources provided mean attack frequencies and durations (from which we calculated times spent in ictal and interictal states).

**Table 2:** **Epidemiological data**

**Epidemiological variable**	***Sub-group***	**China**	**India**	**Russian Federation**	**Zambia**
Migraine prevalence (18–65 years)	*Male*	5.4%	19.4%	12.6%	18.0%
	*Female*	12.6%	32.8%	30.4%	27.1%
Case distribution (attacks per month)	*Low frequency [<3]*	62%	63%	52%	50%
	*High frequency [≥3]*	38%	37%	48%	50%
Mean attacks (per month)	*Low frequency*	1.05	1.05	1.2	0.56
	*High frequency*	5.65	4.59	6.4	3.94
Mean duration of attack (hours)	*All cases*	23.4	13.1	15.0	36.4
Time spent in ictal state (per year)	*Low frequency*	3.4%	1.9%	2.5%	2.8%
	*High frequency*	18.1%	8.2%	13.2%	19.7%

### Estimation of intervention effectiveness

We assessed the impact of acute management of migraine – both with first-line (ASA) and with second-line drugs (sumatriptan or almotriptan) for those not responding to the former – and its combination with a range of prophylactic drugs (for high-frequency cases). For each of these strategies, we also assessed the potential impact of enhanced consumer education and provider training on treatment adherence rates.

We modelled impact as reduction in total time spent in the ictal state. For acute drugs, we used the clinical endpoint of “sustained headache relief” (SHR), defined as reduction in headache intensity from moderate or severe to mild or none (which we assumed was not associated with disability [10] within 2 hours, without recurrence or further medication during 24 hours (Table 1). We assumed baseline pain of migraine was always at least moderate (Table 1 [11]). SHR therefore implied full recovery of the remaining hours of the attack that would have been spent with disability. We assumed that treatment was taken at attack onset (Table 1), so that hours recovered were attack duration minus 2 hours. We obtained SHR values for each acute drug from systematic literature reviews [19-21]. As these were based on clinical trials reporting single doses, we assumed each acute medication was used once per attack (Table 1).

To enable an estimation of real-world effectiveness of prophylactic drugs, data were collected only from trials with a placebo-free baseline period and when fully reported for a minimum of 100 representative and evaluable patients in the active treatment group [22-24]. The median percentage of migraine attacks averted was calculated (Table 3). We assumed prophylaxis would be offered only to the proportion of people with ≥3 migraine attacks/month (Table 1), and derived this proportion for each country from the LTB surveys (Table 2). The potential effect of acute treatment was projected onto attacks not averted by prophylaxis.

**Table 3:** **Efficacy, coverage and adherence values used in base-case analysis**

**Intervention**	**Efficacy**	**Coverage**		**Provider adherence**		**Patient adherence**	
		**Current**	**Target**	**Current**	**Target**	**Current**	**Target**
***Drugs providing sustained headache relief***							
ASA 1,000 mg	39% [21]	80%	90%	100%	100%	80%	90%
		(Zambia 50%)					
Sumatriptan 50 mg	35% [19]	2%	(Zambia 80%)	75%	88%	56%	78%
		(Zambia 1%)					
Almotriptan 12.5 mg	45% [20]	0%		75%	88%	56%	78%
***Drugs averting migraine attacks***							
Propranolol 160 mg	28% [22]	3%	30%	75%	88%	71%	86%
Topiramate 100 mg	40% [23]	1%		75%	88%	60%	80%
Amitriptyline 100 mg	44% [24]	3%		75%	88%	42%	71%

Estimates of efficacy obtained from clinical trials were adjusted better to reflect effectiveness in the real world by reference to treatment coverage (the proportion of people in need of the treatment who receive it) and adherence (Table 3). We assumed ASA had high current coverage (80%) everywhere except in Zambia (50%) (Table 1). We based estimates of triptan coverage on sales data obtained from the Intercontinental Medical Statistics (IMS) database: 2% for sumatriptan (Zambia 1%) and 0% for almotriptan in all countries modelled. We set target coverage at 90% (Zambia 80%) for acute drugs and at 30% (reflecting need limited to high-frequency cases) for prophylactic drugs (Table 3).

To the extent that we could, we based estimates of current patient adherence rates on a systematic literature review. No evidence was found for patient adherence to OTC-drugs in monotherapy, so 80% was used. This was based on assumptions (Table 1) regarding the following components of non-adherence: [I] Not taking the OTC-drugs at all. Conceivably, this is true for 10% of patients covered and thus contributing with 10% to a total percentage of OTC-drug non-adherence. [II] Taking OTC-drugs too late (1 hour or more after onset). We assumed that 10% use it after 1 hour, another 10% use it after 2 hours, another 10% after 3 hours, and another 10% after 4 hours. They are losing an average of 2.5 hours from the possible gain of 16.7 hours (global mean attack duration minus 2 hours). This contributes with 6% (40% of patients * 15% of possible gain) non-adherence. [III] Using OTC-drugs in a too low dose. We assumed that 10% use half of the dose. Of them, probably one half has an effect, so this would contribute with another 5% to the total OTC-drug non-adherence. [IV] We assumed the model could not cope with overusage, so that was ignored. For prescribed drugs (triptans and prophylactics), we used the median current patient adherence rate reported in the literature. This was 56% for triptans [25-30], 71% for propranolol (betablockers) [22,31-34], 60% for topiramate (antiepileptics) [22,24,31,32,34-37], and 42% for amitriptyline [24,31,32,34]. Where combinations of drugs were involved, we modelled the effects of each separately, implying no interaction between adherences to each component. There was no published evidence for provider adherence data. However, there was reason to say it was not 100%. For triptans, we knew some physicians were unduly cost-conscious (and limit supply inappropriately) and that for prophylactics there was under-dosing (both correctable in theory through education). Therefore, we assumed provider adherence to be 75% (Table 1). Target consumer and provider adherence rates were calculated based on the assumption that public education would not improve adherence to 100% but by 50% of the current deficit (Tables 1 and 3) [38-41].

### Estimation of costs

We adopted an “ingredients” approach with a societal perspective, including, as applicable, drug dosage and frequency, primary-care visits, consumer education and provider training. The base year was 2008, and the time horizon used was 10 years of full implementation.

We obtained supplier prices for generically produced drugs (Table 4) from the International Drug Price Indicator Guide for 2008, adjusting to include domestic margin [42]. The cheapest retailer prices for drugs not in the guide (sumatriptan, almotriptan and topiramate) were obtained from the IMS database (third quarter 2007). For sumatriptan (and topiramate in Russia), we used 2014 prices because of price collapses. We imputed prices for countries where drugs were not currently available. For acute medications, we multiplied mean costs per attack by the number of attacks per year in the country population to give the total cost of the intervention per year of implementation.

**Table 4:** **Drug prices (US$) used in base-case analysis**

**Drug**	**Dose**	**Source**	**China**	**India**	**Russia**	**Zambia**
**ASA**	500 mg	International drug price indicator guide	$ 0.004	$ 0.004	$ 0.004	$ 0.004
**Propranolol**	160 mg		$ 0.005	$ 0.005	$ 0.005	$ 0.005
**Amitriptyline**	100 mg		$ 0.006	$ 0.006	$ 0.006	$ 0.006
**Topiramate**	100 mg		$ 0.13	$ 0.03	$ 0.12	$ 0.133
**Sumatriptan**	50 mg	IMS database	$ 0.81	$ 0.11	$ 1.07	$ 0.66
**Almotriptan**	12.5 mg		$ 5.19	$ 5.19	$ 5.19	$ 5.19

We assumed that three-monthly doctor visits were needed for monitoring and prescription of triptans and prophylactics (Table 1). Unit-costs of primary-care services were derived from an econometric analysis of a multinational dataset of hospital costs, using gross national income per capita (plus other explanatory variables) to predict unit costs. The mean duration of a doctor’s visit was defined as 10 minutes (Table 1).

For consumer education, we assumed the number of leaflets needed was 50% of the disease prevalence, and one poster was required per 2,000 of the population (Table 1). We applied WHO-CHOICE default prices to leaflets and posters (and their associated distribution). For provider education, we assumed that one physician per primary health-care centre per year would be trained for one day (Table 1).

## Results

Population-level health effects, costs and cost-effectiveness of the different management strategies are reported for the four countries in Table 5. Results are presented for specified levels of treatment coverage in the target population, first at prevailing rates of patient and provider adherence, then with improved rates of adherence that reflect the addition of consumer education and provider training.

**Table 5:** **Health effects, costs and cost effectiveness of migraine management strategies in China, India, Russian Federation and Zambia**

		**China**							**India**							**Russian Federation**							**Zambia**						
	Target coverage*	**Healthy life years gained per year (per 1 m popn)**		**Cost per year per capita (US$)**		**Cost per healthy life year gained (US$)**		**ICER (US$)**	**Healthy life years gained per year (per 1 m popn)**		**Cost per year per capita (US$)**		**Cost per healthy life year gained (US$)**		**ICER (US$)**	**Healthy life years gained per year (per 1 m popn)**		**Cost per year per capita (US$)**		**Cost per healthy life year gained (US$)**		**ICER (US$)**	**Healthy life years gained per year (per 1 m popn)**		**Cost per year per capita (US$)**		**Cost per healthy life year gained (US$)**		**ICER (US$)**
A. ACUTE MANAGEMENT (NON-SPECIFIC DRUGS)																													
**Simple analgesics (e.g. ASA 1 g)**	90%	530	$	0.02	$	34	$	35	673	$	0.05	$	73	$	75	1223	$	0.07	$	53	$	63	1244	$	0.03	$	24	$	24
With consumer education		597	$	0.03	$	53	$	204	757	$	0.06	$	77	$	105	1376	$	0.19	$	136	$	801	1400	$	0.12	$	85	$	575
B. ACUTE MANAGEMENT (SPECIFIC DRUGS)																													
**Sumatriptan (50 mg)**	90%	152	$	2.14	$	14,061			193	$	1.20	$	6,201			352	$	12.55	$	35,684			358	$	2.62	$	7,330		
With consumer education		212	$	2.16	$	10,159			269	$	1.21	$	4,485			490	$	12.67	$	25,869			498	$	2.71	$	5,442		
With provider training		178	$	2.16	$	12,129			226	$	1.20	$	5,335			410	$	12.64	$	30,804			417	$	2.64	$	6,329		
With consumer education and provider training		248	$	2.17	$	8,762			314	$	1.21	$	3,859			571	$	12.76	$	22,330			581	$	2.73	$	4,698		
**Almotriptan (12.5 mg)**	90%	196	$	5.59	$	28,546			249	$	14.55	$	58,533			452	$	23.38	$	51,728			460	$	8.82	$	19,187		
With consumer education		273	$	5.61	$	20,544			346	$	14.56	$	42,049			630	$	23.51	$	37,332			640	$	8.91	$	13,915		
With provider training		229	$	5.61	$	24,528			290	$	14.56	$	50,187			527	$	23.47	$	44,508			536	$	8.84	$	16,482		
With consumer education and provider training		318	$	5.62	$	17,652			404	$	14.56	$	36,053			735	$	23.60	$	32,121			747	$	8.93	$	11,953		
C. ACUTE STEPPED CARE MANAGEMENT																													
**ASA (1 g) + sumatriptan (50 mg)**	90%	431	$	2.52	$	5,840			547	$	1.62	$	2,964			994	$	15.71	$	15,811			1011	$	2.95	$	2,917		
With consumer education		600	$	2.53	$	4,215			761	$	1.63	$	2,139			1384	$	15.84	$	11,440			1408	$	3.04	$	2,158		
With provider training		503	$	2.53	$	5,033			638	$	1.62	$	2,548			1160	$	15.80	$	13,630			1179	$	2.97	$	2,517		
With consumer education and provider training		700	$	2.54	$	3,633	$	24,271	888	$	1.63	$	1,839	$	11,996	1615	$	15.93	$	9,861	$	65,920	1643	$	3.06	$	1,861	$	12,102
D. PROPHYLAXIS + ACUTE MANAGEMENT																													
**ASA (1 g) + amitriptyline (100 mg)**	30%	112	$	0.18	$	1,649			145	$	0.26	$	1,795			275	$	1.45	$	5,264			305	$	0.24	$	773		
With consumer education		189	$	0.20	$	1,047			245	$	0.27	$	1,098			465	$	1.57	$	3,377			515	$	0.32	$	631		
With provider training		130	$	0.20	$	1,517			169	$	0.26	$	1,565			321	$	1.54	$	4,790			355	$	0.25	$	716		
With consumer education and provider training		220	$	0.21	$	959			286	$	0.27	$	957			543	$	1.66	$	3,059			601	$	0.34	$	573		
**Sumatriptan (50 mg) + amitriptyline (100 mg)**	30%	49	$	0.73	$	14,872			63	$	0.53	$	8,329			121	$	3.95	$	32,633			134	$	1.09	$	8,102		
With consumer education		83	$	0.74	$	8,962			107	$	0.54	$	5,010			205	$	4.07	$	19,902			227	$	1.18	$	5,188		
With provider training		68	$	0.74	$	10,908			88	$	0.53	$	6,049			168	$	4.04	$	24,027			186	$	1.11	$	5,937		
With consumer education and provider training		115	$	0.76	$	6,571			149	$	0.54	$	3,637			284	$	4.16	$	14,644			315	$	1.19	$	3,796		
**ASA + sumatriptan + amitriptyline**	30%	103	$	0.80	$	7,740			133	$	0.73	$	5,467			248	$	4.55	$	18,319			275	$	1.20	$	4,362		
With consumer education		174	$	0.81	$	4,657			226	$	0.74	$	3,273			420	$	4.67	$	11,128			465	$	1.29	$	2,773		
With provider training		143	$	0.81	$	5,668			185	$	0.73	$	3,961			345	$	4.64	$	13,449			382	$	1.22	$	3,191		
With consumer education and provider training		241	$	0.82	$	3,409			313	$	0.74	$	2,371			583	$	4.76	$	8,166			645	$	1.31	$	2,026		

*Target coverage for acute management in Zambia set at 80%.

ICER=Incremental cost-effectiveness ratio. All interventions without an ICER value are 'dominated’.

### Intervention effectiveness

The population-level health impact of these management strategies, in terms of providing SHR or reducing the frequency of migraine attacks, is quite considerable. For example, and compared to no treatment, acute management with simple analgesics such as ASA annually generates 530 extra HLYs per million population in China and over 1,200 HLYs in Zambia and the Russian Federation; this is as a result of the reduced amount of time spent in a (highly disabling) ictal state. Additional health gain (of an estimated 12.5%) could be realised by enhancing consumer education and therefore adherence to such non-specific drug treatment. The strategy associated with the greatest population-level health gain was acute, stepped-care management using ASA plus sumatriptan for non-responders, together with consumer education and provider training (across the four countries, 700–1,600 HLYs gained per one million population). A combination of acute management and prophylaxis (with amitriptyline) also produces sizeable health benefits, but produces less overall gain in the population because of lower expected coverage and applicability (a sub-population of high frequency migraine cases). Amitriptyline was used in the main analysis because it is considerably more effective than propranolol (Table 3) and also far cheaper than topiramate (Table 4).

### Intervention cost and cost-effectiveness

The annual cost of different management strategies varies enormously, from just a few US cents up to US$ 5–25 per capita population (for almotriptan). This is essentially driven by the price of drugs (over US$ 5 per 12.5 mg tablet of almotriptan, compared to < 1 US cent for a 1,000 mg dose of ASA). For sumatriptan, the price (and therefore overall cost of treatment) is more variable: for example it is about ten times higher in Russia than in India. Evidently, the addition of consumer education and provider training strategies increases the cost of care, but only to a fixed and small degree (of 1–2 US cents per head of population).

Dividing the total cost of each intervention by its associated health benefit provides a measure of its cost-effectiveness, relative to a situation of no treatment. Table 5 shows that the cost per HLY gained ranges from less than US$ 100 (for acute management with simple analgesics) to thousands or even tens of thousands of US dollars (for treatment of analgesic non-responders with triptans). By far the most cost-effective strategy is acute management with simple analgesics (ranging between US$ 24–73 per HLY gained across the four countries); adding in consumer education and thereby improving adherence has a small upward influence on cost-effectiveness ratio; compared to no treatment at all, this strategy falls below US$ 150 per HLY gained, but compared to use of simple analgesics without consumer education, the incremental cost to be paid in order to obtain one extra HLY rises to US$ 200–800. Beyond that, the aforementioned stepped-care strategy using both specific and non-specific drugs as well as consumer education and provider training would further increase population-health gain but at an incremental cost that is expected to fall outside national thresholds for value for money in the health sector (that is, each additional HLY comes at an extra cost of many times the average income per person). Finally, combining prophylaxis (with amitriptyline) and acute management (with ASA) presents a favourable ratio of cost to effect when compared to no treatment, particularly if accompanied by consumer education and provider training (below US$ 600 per HLY gained in Zambia, and below US$ 1,000 in China and India); in incremental cost-effectiveness terms, however, it is ‘dominated’ by the superior cost-effectiveness profile of acute management with simple analgesics alone.

### Sensitivity and uncertainty analyses

A series of sensitivity and uncertainty analyses were carried out in order to examine the impact of plausible levels of variability around baseline estimates. The results of these analyses demonstrate that, even after allowing for this variability, acute management with simple analgesics (with or without consumer education) continues to be easily the most cost-effective strategy for reducing the burden of migraine in lower middle- and middle-income countries. We assessed a) the impact of changes in underlying analytical choices (such as whether to discount health gains or not), b) the influence of specific input parameters on costs and effects (such as drug prices and disability weights), and c) the potential variability around total costs and effects of different interventions.

Baseline results did not employ discounting or age-weighting of health benefits. Discounting health gains over time (by a factor of 3%) increased average cost-effectiveness ratios by 18%; application of an age-weighting function – which places a higher value on health gains in middle aged groups and less for the old and young – on top of discounting has a negligible impact on baseline cost-effectiveness results (<5%).

The most sensitive price in the analysis was sumatriptan. Reducing the applicable price of sumatriptan in each country by 50% has a sizeable impact on the cost and cost-effectiveness profile for this drug (reducing baseline cost-effectiveness ratios [CERs] by 30-40%), but not enough to bring it close to simple analgesics.

Concerning disability weights, there was a question of what disability weight to use – if any – for the interictal state; accordingly we assessed the impact on health effects and cost-effectiveness ratios of using no disability weight and a higher disability weight (0.03), compared to the baseline value of 0.01. Use of a zero disability weight resulted in slightly higher health effects (5%) and therefore marginally better cost-effectiveness ratios (4% lower). The higher disability weight led to less overall health gain (7%) and correspondingly worse cost-effectiveness ratios (8% higher).

**Figure 1:**
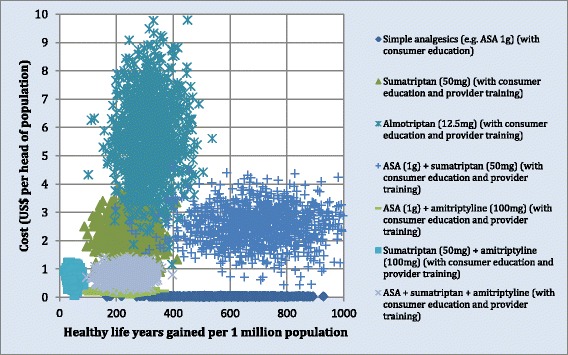
**Uncertainty cloud graph for migraine interventions, China.**

In order to assess the inherent uncertainty around (point) estimates of total intervention costs and health effects, a subset of intervention strategies were entered into an analytical software package (MCLeague), which performs a probabilistic uncertainty analysis using Monte Carlo simulation (1,000 runs were made, using a truncated normal distribution). A subset was chosen for the sake of being able to visualise the results in cloud graph format. The seven selected interventions were ASA + consumer education and all other drug interventions that included consumer education and provider training (since the latter gave better cost-effectiveness ratios than intervention scenarios without one or both of these additional components). We used a coefficient of variation of 0.2 for effects and 0.25 for costs. The graphical results presented in Figure 1 relate to China and demonstrate that, even after allowing for this variability, the average cost-effectiveness ratios of interventions for ASA + consumer education do not overlap appreciably with other interventions, confirming its clear superiority in cost-effectiveness terms. It also shows that after allowing for uncertainty, the stepped-care strategy using both specific and non-specific drugs as well as consumer education and provider training is the next most cost-effective strategy after ASA + consumer education, but incurs significantly greater costs to achieve the same level of population health gain. Similar results pertain to the other countries in the analysis.

## Discussion and conclusions

Acute treatment of migraine attacks with simple analgesics (in this analysis, ASA) generated a whole year of healthy life for less than US$ 100. This means that it is among the most efficient interventions to improve population health. The context in which this finding must be set is that migraine is the third most prevalent disease in the world, and the seventh highest specific cause of global disability [2,43]. In other words, evidence-based interventions for migraine should have as much a claim on scarce health resources as other chronic, non-communicable conditions that impose a substantial burden on societies, and a greater claim than many. The potential for health gain in society is huge. In three of the countries in question (Russia [15], India and Zambia [data from LTB, submitted for publication]), the 1-year prevalence of migraine (range 20.3-25.6%) is well-above the global mean of 14.7% [2]. Not only is the cost of this intervention low, but also there is little requirement for health-service infrastructural support that might be a barrier to its implementation or prevent widespread coverage.

Simple analgesics are not of course the complete answer to migraine: they have limited efficacy (39% SHR was built into the model, implying no benefit for 61%). In migraine management following European guidelines, prescription drugs are called in as reinforcements only when needed, in a stepped-care paradigm [6,7]. Prescribed drugs, as second-line in acute treatment or for prophylaxis, are less cost-effective in our model but not necessarily with an unfavourable ratio of cost to effect when compared with many other interventions aimed at improving population health. We showed that training primary care doctors in the management of migraine is likely to increase the cost-effectiveness of drugs on prescription. We further showed that the incremental health benefits obtained from adding educational programmes were achieved at acceptable incremental costs. Even if marginally less cost-effective, strategies with educational programmes may be worth investing in, since the absolute health-gain is higher with their inclusion. Incorporating the effects of consumer education (posters and leaflets in pharmacies) on coverage was a major strength of our model.

These are entirely new findings. There is no earlier evidence on the cost-effectiveness of OTC drugs alone for migraine even though, worldwide, the great majority of people with headache are primarily self-treating [1]. Efficacy data for prophylactic drugs, mainly from older clinical trials, relate to a change from a placebo run-in period, which underestimates their real-world effect. We selectively used data from trials with a placebo-free run-in period to avoid this. Furthermore, we modelled the impact of non-adherence to provide more realistic estimates. The finding that amitriptyline was more likely to be cost-effective than propranolol is concordant with that of Yu et al. 2010 [4]. Of the few other, earlier CEAs of prophylactic migraine drugs, all were restricted to antiepileptic drugs [44-46].

There are inherent limitations in the study. The population and costing models rest upon a series of best estimates, including the expected patterns of resource use and, perhaps most importantly, estimates of intervention efficacy. Those drawn from trials were derived in western countries, with uncertain application to the countries in question. Not including paracetamol was a limitation forced on us because the only evidence available was from 42 highly atypical US patients, from which we felt unable to extrapolate to these countries [47]. Even though the indirect costs of migraine dwarf the direct costs, productivity gains and time costs were not taken into consideration because no internationally agreed approach is yet available to measure these satisfactorily [5].

Despite these limitations, the study has provided information that should greatly assist regional health-policy makers, in rich and poor countries alike, in allocating fixed health budgets between interventions and between health sectors in order to maximize health in society. What are needed now are CEAs on interventions for headache disorders more broadly, rather than migraine alone, which in real life is not treated in isolation. These include structured headache services, ideally provided at national level, with quality evaluation, and particularly interventions with a substantial educational element [48-50]. Many such initiatives are being pursued by LTB within the Global Campaign against Headache [17].

## Abbreviations

ASA: Acetyl salicylic acid
BRIC: Brazil, Russia, India and China
CEA: Cost-effectiveness analysis
CER: Cost-effectiveness ratio
CHOICE: Choosing Interventions that are Cost-Effective
DW: Disability weight
GBD: The global burden of disease study
HLY: Healthy life years
ICER: Incremental cost-effectiveness ratio
IMS: Intercontinental medical statistics
LTB: Lifting the burden
OTC: Over-the-counter
SHR: Sustained headache relief
WHO: World Health Organization
YLD: Years of healthy life lost to disability
